# Time and Age Trends in Free Sugar Intake from Food Groups among Children and Adolescents between 1985 and 2016

**DOI:** 10.3390/nu12010020

**Published:** 2019-12-20

**Authors:** Ines Perrar, Alena M. Schadow, Sarah Schmitting, Anette E. Buyken, Ute Alexy

**Affiliations:** 1 Institute of Nutritional and Food Sciences-Nutritional Epidemiology, University of Bonn, Donald Study Dortmund, Heinstück 11, 44225 Dortmund, Germany; iperrar@uni-bonn.de (I.P.); alena.schadow@web.de (A.M.S.); schmitting.sarah@web.de (S.S.); 2 Institute of General Practice and Family Medicine, Faculty of Medicine, Ruhr-University Bochum, Universitätsstraße 150, 44801 Bochum, Germany; 3 Institute of Nutrition, Consumption and Health, Faculty of Natural Sciences, Paderborn University, Warburger Straße 100, 33098 Paderborn, Germany; anette.buyken@uni-paderborn.de

**Keywords:** free sugar, trends, children, adolescents, food groups

## Abstract

Trend analyses suggest that free sugar (FS) intake—while still exceeding 10%E—has decreased among German children and adolescents since 2005, yet that intakes may shift from sugars naturally occurring in foods to added sugars as children age. Thus, we analysed time and age trends in FS intake (%E) from *food groups* among 3–18 year-olds (1985–2016) using 10,761 3-day dietary records from 1312 DONALD participants (660 boys, 652 girls) by use of polynomial mixed-effects regression models. Among girls, FS from *sugar & sweets* decreased from 1985 to 2016 (linear trend *p* < 0.0001), but not among boys (*p* > 0.05). In the total sample, FS intake from *juices* increased until 2000 and decreased since 2005 (linear, quadratic trend *p* < 0.0001). FS from *sugar sweetened beverages (SSB)* decreased non-linearly from 1985 to 2016 (girls: linear, quadratic, cubic trend *p* < 0.0001; boys: linear, quadratic, cubic trend *p* < 0.02). Younger children consumed more FS from *juices* than older ones, who had a higher FS intake from *SSB*. FS intake from *sugar & sweets* increased until early adolescence and decreased afterwards. Since *sugar & sweets* represent the main source of FS intake and the source with the least pronounced decline in intake, public health measures should focus on these products.

## 1. Introduction

A high dietary sugar intake is thought to contribute to the development of several diseases such as dental caries [1], overweight and obesity [2,3,4,5], cardiovascular diseases [6,7,8] or metabolic syndrome [9]. With the recent shift towards food-based recommendations, evidence has emerged regarding the relevance of specific food source of sugar for health outcomes [10,11,12,13,14,15,16]. In particular, a high intake of sugars from liquid sources may be detrimental for health: Due to a lack of satiety [17] and incomplete compensation of liquid calories [10], a high sugar intake from SSB promotes weight gain and the development of overweight [3,12,13,18] or diabetes [11,15]. Since pure juices can have sugar and energy contents similar to SSB [16], intakes of juices are also of public health relevance.

Within the term “free sugar” [15] the World Health Organization (WHO) considers “all monosaccharides and disaccharides added to foods by the manufacturer, cook or consumer, plus sugars naturally present in honey, syrups and fruit juices” [19]. In addition, the UK Scientific Advisory Committee on Nutrition (SCAN) has recently recommended extending the term of FS to fruits and vegetables purees and pastes and similar products in which the cell structure was destroyed during processing [20,21]. International and national organizations recommend limitting FS intake to a maximum of 10% of energy intake (%E) [20,21,22,23]. However, this recommendation as well as some possible health consequences of high sugar intake are controversially discussed, in particular by the food industry [24,25].

As the sweet preference is higher in childhood and adolescence than in adulthood [26,27,28], these ages are particularly vulnerable to the health consequences of high sugar intakes. In fact, recent publications indicated a high intake of FS among European children and adolescents, exceeding the 10%E limit to a larger extent than among adults [29,30,31]. Such a high sugar intake may be of particular relevance, as adolescence is suggested to be a “critical period” for the development of various diseases in later life [32,33,34,35] and dietary patterns have been shown to track into adulthood [36].

However, dietary habits may change with time and with increasing age, as the sweet preference is already decreasing from childhood to adolescence [26,27,28]. Recent trend analyses among German children and adolescents from the DONALD (Dortmund Nutritional and Anthropometric Longitudinally Designed) study cohort showed a decline in free sugar intake (in %E, model adjusted among others for overweight status) since 1985, most notably since 2010 [30]. Age trends in total, added and free sugar intake suggest a shift from those sugars naturally occurring in fruits, milk, and juices to those added to foods and beverages, e.g., sweets and sweetened beverages from 3 to 18 years of age [30]. However, as daily FS intake still exceeded 10%E over the complete study course and age range [30], initiatives to further reduce FS intake among children and adolescents are needed.

For the development of tailored public health measures for FS reduction, data on FS food sources preferred by children and adolescents are crucial. Therefore, we analysed time and age trends in food group sources of FS from three decades (1985–2016) among children and adolescents aged 3–18 years, using data from 3-day weighed dietary records from the DONALD study.

## 2. Materials and Methods

### 2.1. Study Sample

The DONALD study is an ongoing, open cohort study conducted in Dortmund, Germany, which started to collect information on diet, growth, development and metabolism of healthy children and adolescents in 1985. Since then, 35–40 infants are newly recruited every year. Eligible are healthy German infants (i.e., infants free of diseases affecting growth and/or dietary intake), whose parents are willing to participate in a long-term study and of whom at least one has sufficient knowledge of the German language. The participants are first examined at the age of 3 months and return for three more visits in the first year, two in the second year and thereafter annually until young adulthood. In the first study years, approximately 300 participants >2 years old were also recruited. Yearly examinations include 3-day weighed dietary records, anthropometric measurements, collection of 24-h urine samples (starting at age 3–4 years), interviews on lifestyle and medical examinations. Parental examinations (anthropometric measurements, lifestyle interviews) take place every four years. Further details on the study have been described elsewhere [37,38]. The study was approved by the Ethics Committee of the University of Bonn according to the guidelines of the Declaration of Helsinki, and all examinations are performed with parental and later on, children’s written consent.

At the start of the dataset compilation for the current investigation (October 2017) 17,107 records were available from the DONALD database. Incomplete records (<3 days, *n* = 176) were excluded as well as records from <3 years old (*n* = 5618) or >18 years old (*n* = 421) participants and records carried out after December 2016 (*n* = 131). For the present evaluation, we hence analysed 10,761 complete dietary records from 1312 DONALD study participants (660 boys, 652 girls). Per participant, between one (*n* = 153, 11.7%) and sixteen (*n* = 184, 14.0%) dietary records [median (Q1; Q3): 8 (3; 13)] were available.

### 2.2. Nutrition Assessment

Dietary intake in the DONALD study is assessed using 3-day weighed dietary records. All foods and beverages consumed by the child, as well as leftovers, are weighed and recorded over three consecutive days by the parents or by the older participants themselves, with the use of electronic food scales (±1 g). The participants choose the day of the beginning of dietary recording within a given period of time. When exact weighing is not possible, household measures (e.g., spoons, cups) are allowed for semi-quantitative recording. Information on recipes (ingredients and preparation) and on the types and brands of food items consumed is also requested. Medication and dietary supplement use are also recorded but were excluded from this analysis. A trained dietitian checks the dietary records for accuracy and completeness. Subsequently, energy and nutrition intakes are calculated using our continuously updated in-house nutrient database LEBTAB [39]. The composition of staple foods is based on the German food composition tables BLS 3.02. Energy and nutrient contents of commercial food products, i.e., processed foods and ready-to-eat-meals are estimated by recipe simulation using labelled ingredients and nutrient contents. FS was defined according to the definition by SACN [20,21], including added sugars plus sugars from fruit juices, vegetable juices, juice spritzers and smoothies. Energy, nutrient and food group intakes were calculated as individual means of three days of recording.

### 2.3. Definitions of Outcome Variables

**Figure 1:**
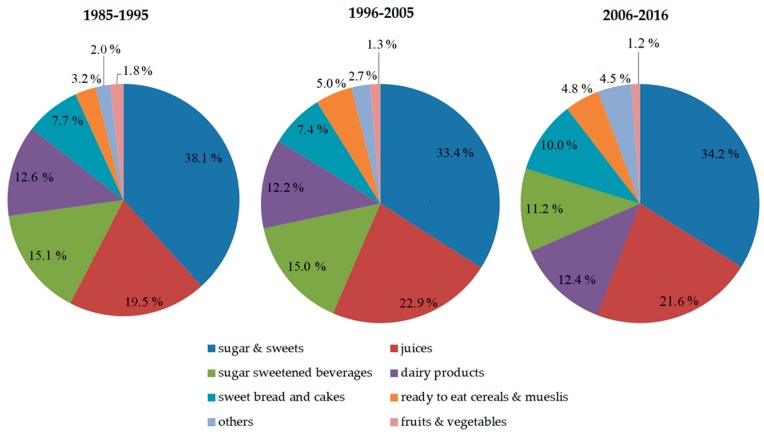
Percentages of FS intake from food groups, stratified by time period (1985–1995, 1996–2005, 2006–2016).

FS intake from the following food groups were examined as outcome variables: *sugar & sweets, dairy products, juices, sugar sweetened beverages [SSB], sweet bread & cakes, ready to eat breakfast cereals [RTC], others*. The intake of FS from fruits & vegetables was very low (<2% of FS intake, Figure 1), so we decided not to carry out any trend analyses for this outcome. Definitions of the eight food groups are shown in Table 1. Sugar intakes were calculated as the percentage of total daily energy intake (%E) to enable comparison of sugar intake between different age groups and energy intake levels.

### 2.4. Assessment of Potential Confounding Factors

For this analysis, the following characteristics were considered as potentially confounding factors: sex (boy/girl), overweight status (yes/no), number of weekdays per 3-day record (1/2/3), maternal overweight (yes/no), high maternal educational status (yes/no), maternal employment (yes/no). Height and weight are measured by nurses according to standard procedures with the participants dressed in underwear only and barefoot. From the age of 2 years onwards, standing height is measured to the nearest 0.1 cm using a digital stadiometer (Harpenden, Crymych, UK). Body weight is measured to the nearest 100 g using an electronic scale (Seca 753E; Seca Weighing and Measuring System). Body mass index (BMI [kg/m^2^]) was calculated as the body weight (kg) divided by the square of the body height (m). Overweight was defined according to International Obesity Task Force’s (IOTF) BMI cutoff values for children and adolescents [40,41]. Maternal body weight and height are measured with the same equipment as for the participants. Maternal overweight was defined as a BMI ≥ 25 kg/m^2^. High maternal educational status (≥12 years of schooling) and maternal employment are inquired with a standardized questionnaire. For missing values, the respective median of the total sample was used (*n* = 38 for maternal overweight, *n* = 5 for maternal educational status).

### 2.5. Statistical Analysis

The statistical analyses of the present evaluation were performed using SAS^®^ procedures (version 9.4; Cary, NC, USA). The significance level was set at *p* < 0.05. Characteristics in the tables are presented as medians with their interquartile range or frequencies and percentages. For the presentation of dietary descriptive data in tables, participants were stratified according to time periods (1985–1995, 1996–2005 and 2006–2016) and (age groups: >2.5–<5.5, ≥5.5–<10.5, ≥10.5–<13.5 and ≥13.5–<18.5 years, respectively).

Time and age trends in FS (%E) were analysed using polynomial mixed-effects regression models including both fixed and random effects (PROC MIXED in SAS^®^). In cases of significant interactions between the time or age and sex (sex × age, sex × time), stratified analyses were performed. Time and age—continuously in years—were the principle fixed effects of the models. The first included record in this evaluation was considered the baseline time, i.e., time = 0. Therefore, time ranged between 0 and 31 years. Quadratic and cubic terms for age (age^2^, age^3^) and time (time^2^, time^3^) as well as a combination of the linear time and age variable (age × time) were considered as additional explanatory variables if they improved the fit statistics [Akaike information criterion (AIC)] by more than two points or significantly predicted the respective outcome [42]. A linear trend reflects a constant increase or decrease in the respective outcome variable over the years or with age. Quadratic and cubic trends indicate that the magnitude of the trend changes over time or with age. A repeated statement was considered in order to account for the lack of independence between repeated measures from the same person. Random effects were considered to allow variation between individuals and families with respect to the initial level (intercept) as well as linear, quadratic and cubic age trends of the respective outcome. The AIC was also used to select the covariance structure that best describes the variances and covariances of the initial level, the linear and quadratic trend among persons, and the covariance structure that best describes the correlated nature of the repeated measurements. Covariables that were considered in the final models either (1) modified regression coefficients in the basic models by ≥10% (2), had a significant and independent association with the outcome variable, or (3) led to an improvement of the AIC by more than two points [43].

The single effect estimates of polynomial models cannot be interpreted, i.e., if the analyses render significant results for a combination of linear, quadratic, and cubic trends, the single beta values do not reflect the true time and age trends. Hence, figures were designed to illustrate the predicted trends. These figures show the predicted FS intake from the individual food groups resulting from the polynomial mixed-effects regression models over the course of the study period for different age groups (3/4 years, 5/6 years, 7/8 years, 9/10 years, 11/12 years, 13/14 years, 15/16 years and 17/18 years). Thus, the course of the curves illustrates the time trend and the vertical differences between the curves for different ages indicate the age trend.

Records were considered as under-reported when the total energy intake (TEI) was inadequate in relation to the estimated basal metabolic rate (BMR) (according to age- and sex-specific equations of Schofield [44]), using pediatric cutoffs [45]. This procedure resulted in 835 (7.8%) records with under-reporting. Under-reported records were not excluded from the analyses, as this procedure only identifies under-reported energy intake, but no selective under-reporting of food groups [46] or sugar intake [47]. In addition, participants with high energy requirements (e.g., due to high physical activity levels), who may have under-reported, could not be identified [48]. Instead, sensitivity analyses excluding records, which were identified as under-reported, were done.

To check whether differences in total daily energy intakes arising from differences in age, sex or physical activity level of the participants as well as changes in total daily energy intake over time may have confounded the results, additional sensitivity analyses were performed, additionally accounting for total daily energy intake (kcal).

## 3. Results

### 3.1. Sample Characteristics

Overall 10,761 3-day weighed dietary records from 1312 DONALD study participants were analysed. Approximately half of the participants were female. Participants’ overweight status and maternal characteristics reflect the high socioeconomic status (SES) of DONALD participants (Table 2). Only 12.3% of the participants were overweight at the time of measurement.

For the total study sample, mean FS intake was 17.1 ± 6.6%E. Percentages of FS intake from food groups of the total sample are illustrated in Figure 1, stratified by three time periods (1985–1995, 1996–2005 and 2006–2016). *Sugar & sweets* constitute the largest proportion of FS intake in all time periods (38.1%, 33.4% and 34.2%, respectively), followed by *juices* (19.5%, 22.9% and 21.6%, respectively). While in the years 1985–1995 and 1996–2005 *SSB* (15.1% and 15.0%, respectively) they were the third largest source of FS, in 2006–2016, *dairy products* were the third largest source of FS (12.4%). Dietary characteristics stratified by time periods are shown in Table 3 and stratified by age groups are shown in Table 4.

### 3.2. Time and Age Trends

**Figure 2:**
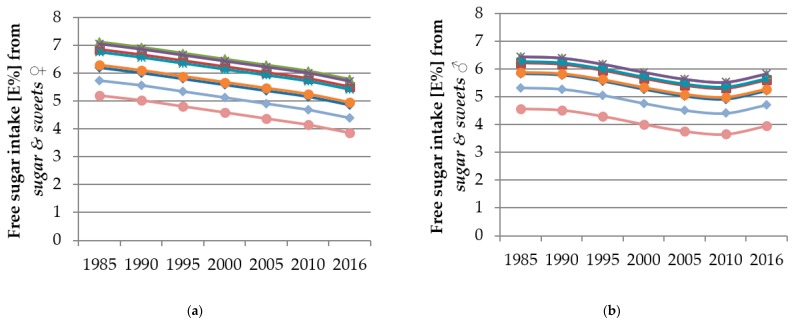
Time and age trends in FS intake from *sugar & sweets* among girls (**a**) and boys (**b**) of 10,761 dietary records of 660 male and 652 female DONALD study participants (3–18 years) between 1985 and 2016, predicted by polynomial mixed-effects regression models (see Table 5) (blue rhombi 3/4 year-olds, red squares 5/6 year-olds, green triangles 7/8 year-olds, purple crosses 9/10 year-olds, turquoise stars 11/12 year-olds, orange circles 13/14 year-olds, light blue rhombi 15/16 year-olds, pink circles 17/18 year-olds).

**Figure 3:**
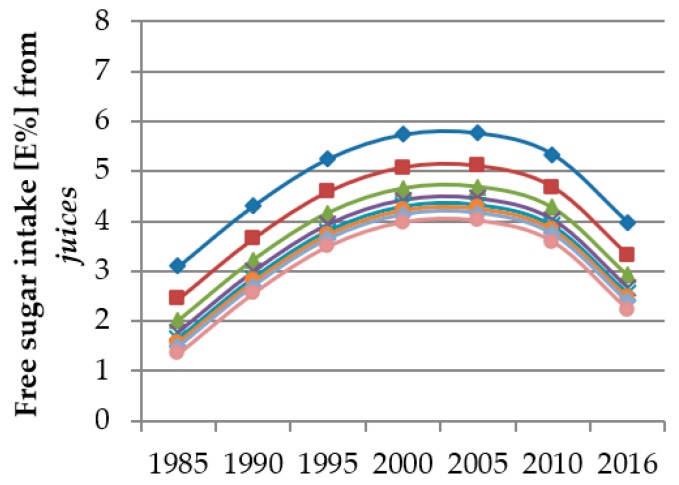
Time and age trends in FS intake from *juices* of 10,761 dietary records of 1312 DONALD study participants (3–18 years) between 1985 and 2016, predicted by polynomial mixed-effects regression models (see Table 5) (blue rhombi 3/4 year-olds, red squares 5/6 year-olds, green triangles 7/8 year-olds, purple crosses 9/10 year-olds, turquoise stars 11/12 year-olds, orange circles 13/14 year-olds, light blue rhombi 15/16-year-olds, pink circles 17/18 year-olds).

**Figure 4:**
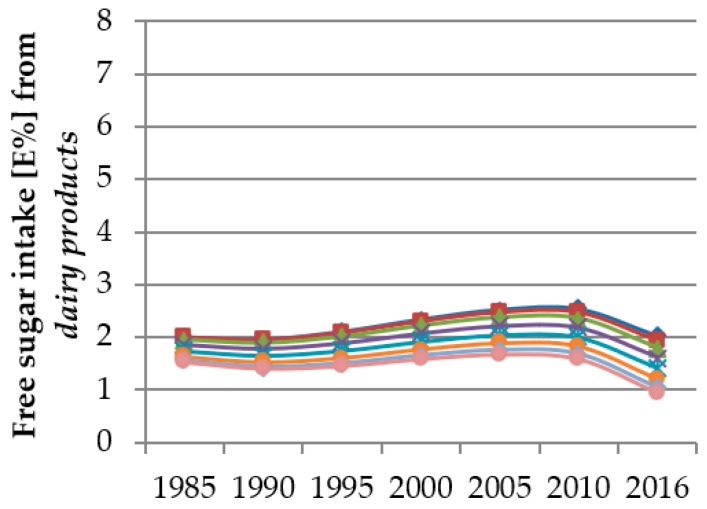
Time and age trends in FS intake from *dairy products* of 10,761 dietary records of 1312 DONALD study participants (3–18 years) between 1985 and 2016, predicted by polynomial mixed-effects regression models (see Table 5) (blue rhombi 3/4 year-olds, red squares 5/6 year-olds, green triangles 7/8 year-olds, purple crosses 9/10 year-olds, turquoise stars 11/12 year-olds, orange circles 13/14 year-olds, light blue rhombi 15/16 year-olds, pink circles 17/18 year-olds).

**Figure 5:**
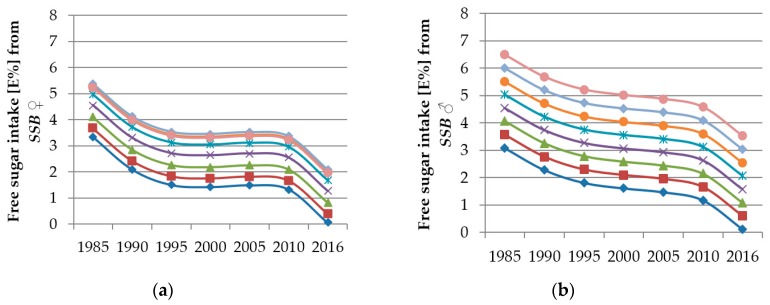
Time and age trends in FS intake from *SSB* among girls (**a**) and boys (**b**) of 10,761 dietary records of 660 male and 652 female DONALD study participants (3–18 years) between 1985 and 2016, predicted by polynomial mixed-effects regression models (see Table 5) (blue rhombi 3/4 year-olds, red squares 5/6 year-olds, green triangles 7/8 year-olds, purple crosses 9/10 year-olds, turquoise stars 11/12 year-olds, orange circles 13/14 year-olds, light blue rhombi 15/16 year-olds, pink circles 17/18 year-olds).

Results of the time and age trend analyses from the polynomial mixed-effects regression models for FS intake from *sugar & sweets*, *juices*, *dairy products* and *SSB* are shown in Table 5 and displayed in Figure 2, Figure 3, Figure 4 and Figure 5. Changes in FS intake from *sweet breads & cakes*, *RTC* and *others* with age or during the observation period were negligible (see Supplementary Table S1 and Figures S1–S3).

With respect to time trends, among girls, FS intake from *sugar & sweets* decreased between 1985 and 2016 continuously (Figure 2a, linear trend *p* < 0.0001). Among boys, FS intake from *sugar & sweets* did not change significantly over the study period.

Among the total sample, FS intake from *juices* increased between 1985 and 2000, remained constant until 2005 and subsequently decreased until 2016, reaching intake levels comparable to those seen in 1990 (linear and quadratic trend *p* < 0.0001) (Figure 3).

FS intake from *dairy products* increased slightly between 1985 and 2010 and decreased thereafter (quadratic trend *p* = 0.0009, cubic trend *p* < 0.0001) (Figure 4). The test on interaction between time and age was significant only in the model of FS intake from *dairy products* (*p* = 0.0489). Therefore, we included the term age × time in the model. This leads to the fact that the extent of time trends in FS intake from dairy products was dependent on age (Figure 4).

Among girls and boys, significant time trends in FS intake from *SSB* were observed (girls: linear, quadratic, cubic trend: *p* < 0.0001; boys: linear trend *p* = 0.0028, quadratic trend *p* = 0.0165, cubic trend *p* = 0.0135): FS intake from *SSB* among girls decreased from 1985 to 1995, stagnated until 2010 and decreased again thereafter (Figure 5a). FS intake from *SSB* among boys decreased continuously (Figure 5b).

In terms of age trends, FS intake from *sugar & sweets* changed with age among girls and boys (girls: linear and quadratic trend *p* < 0.0001, cubic trend *p* = 0.0033; boys: linear and quadratic trend *p* < 0.0001). The oldest girls and boys, followed by the 15/16 year-olds, had the lowest FS intakes from *sugar & sweets* (Figure 2a,b). The 7/8 and 9/10 year-olds had the largest FS intakes from *sugar & sweets*, yet only slightly higher than intake levels among the 5/6 and 11/12 year-olds (Figure 2a,b).

The youngest children, followed by the 5/6 year-olds, had the highest FS intakes from *juices* (linear trend *p* < 0.0001, quadratic trend *p* = 0.0009, cubic trend *p* = 0.0082). Differences among the other ages were negligible (Figure 3).

Younger participants also consumed more FS from *dairy products* than older ones (quadratic trend *p* = 0.0207, cubic trend *p* = 0.028). Due to the significant interaction between time and age in this model, differences between the ages were hardly discernible at the beginning of the observation period and became more obvious at the end (Figure 4).

In boys, younger participants had lower FS intakes from *SSB* than older participants (linear trend *p* < 0.0001) (Figure 5b). A similar trend was observed among girls. However, 15/16 year-olds had a larger FS intake from *SSB* than 17/18 year-olds (cubic trend *p* = 0.0211) (Figure 5a). In addition, age differences in FS intake from *SSB* among girls were not as clear as among boys.

Sensitivity analyses excluding under-reported records as well as including total daily energy intake as covariable, yielded similar results for time and age trends.

## 4. Discussion

The present study identified time and age trends in FS intake from *sugar & sweets*, *dairy products*, *fruits & vegetables*, *juices* and *SSB*, which are relevant for the implementation of public health measures to reduce FS intake among children and adolescents. The observed decline in FS intake from *sugar & sweets*, *juices*, *dairy products* and *SSB* over the study course provides additional insight into the nature of the time trends observed in previous analyses among the present study sample: Total FS intake increased between 1985 and 2005 and decreased, most notably since 2010 [30]. The increase in FS intake from *juices* between 1985 and 2005 contributes largely to the increase in total FS intake in this time period. While FS intake from *juices* and *SSB* as well as FS from *sugar & sweets* among girls started to decline since 2005, FS intake from *dairy products* only decreased from 2010 onwards, hence explaining the notable decrease in total FS intake since 2010.

Furthermore, we observed differences in the extent of FS decline between the investigated food groups. The decline in FS intake from *sugar & sweets* was the least pronounced. In contrast, the recent decline in FS intake from *juices* was the most pronounced, followed by FS intake from *SSB*, which may reflect an increasing awareness of health risks associated with liquid sugar sources [11,12,13,15,18,49]. This might be due to the comparably high SES of our study sample. A further explanation for these results might be a *SSB* specific under-reporting, since *SSB* intake is increasingly discussed to promote diseases such as overweight and obesity [2,3,4,5]. For the same reason, participants could consciously abstain from *SSB* during the dietary record days and thus, eat and record less FS from *SSB* than usual. Although we cannot preclude that the decreasing trend in FS intake from *SSB* partly reflect sugar specific misreporting [47], our sensitivity analyses do not support the notion of bias from general under-reporting since results for time and age trends were similar when we excluded under-reported records.

At the time of the decline of total FS intake or FS intake from food groups, e.g., from *SSB*, *juices* or *sugar & sweets* in Germany, measures to reduce sugar intake were non-systematic and directed at the individuals (e.g., education initiatives in kindergarden and schools), hence, the cause of the observed decrease of FS intake from many food groups in recent years is unclear. However, total FS intake still markedly exceeded the 10%E limit, set by the WHO, across all age groups and time windows [30]. Our data suggest that public health strategies focusing on FS from *sugar & sweets* are needed to reduce intake from these sources among children and adolescents. To date, only voluntary reformulation measures have been implemented in Germany (since 2019) and will be complemented by voluntary front-of-package logos, i.e., the NutriScore in due course [50]. However, the current reformulation strategy explicitly excludes *sugars & sweets* [51,52] and a voluntary introduction of the NutriScore is unlikely to affect the choice of *sugar & sweets*. Hence, additional measures—ideally targeted at *sugar & sweets*—will be necessary.

Time trends in FS intake from *SSB* differed depending on the sex of the participants: the declining trend in FS intake from *SSB* was less pronounced among girls than among boys. This is probably due to the fact that boys generally consume more SSB than girls, which was recently confirmed in a representative study among children and adolescents in Germany [53,54]. Hence, FS intake from *SSB* may have decreased faster among boys than among girls.

To our knowledge, no time trend analyses in FS intake from food groups among other young study populations existed up to now. Welsh et al. investigated time trends in added sugars (AS, i.e., FS minus sugar from juices) intake from different food groups among young participants (2–17 years) from the National Health and Nutrition Examination Survey (NHANES) in the U.S. [55]. In line with our study results, significant linearly decreasing time trends in AS intake from both sweets and regular sodas were observed between 1999/2000 and 2007/2008 [55]. Studies among European children only examined time trends in the intake of food groups, but not sugar intake levels: Stahl et al. (2009) compared food intake among 6–17 year-olds between two cross-sectional representative German surveys, the German National Food Consumption Study (Nationale Verzehrsstudie, NVS) from 1985–1988 (*n* = 2265) and the nutrition module ‘EsKiMo’ of the German Health Interview and Examination Survey for Children and Adolescents (KiGGS) from 2003 to 2006 (*n* = 2506) [56]. In this study, children consumed significantly more confectionery/jam/sugar and non-alcoholic beverages (juices and SSB) in 2003–2006 than children in 1985–1988 [56]. In a comparison between the first (2003–2006) and the second KiGGS (2014–2017) survey, the daily consumption of confectionary [53] and SSB [53,54] decreased. Our findings also point to an increasing *juice* intake between 1985 and 2006, while the intakes of *sugar & sweets* and *SSB* already decreased between 1985 and 2006. Differences in time trends between these studies may be explained by different populations, methodologies and statistical tests. Furthermore, it must be kept in mind that we examined FS intake from food groups, while the other studies investigated absolute intake amounts. A decline in FS intake from food groups may not be solely attributable to a decline consumed amounts. It could also reflect product reformulation during this time period, since food manufacturers change the recipe of their products, e.g., the sugar content, over time. Due to its continuously updated in-house nutrient database LEBTAB [39] the DONALD study is probably the only study that can take this change into account: if the recipe of a product was changed, resulting in different nutrient and energy contents of the product, a new entry was added to LEBTAB, whilst the entry for the product with the old recipe was marked and retained in the database. Thus, our analyses account for recipe changes in our time trends.

This fact also allows us to investigate differences in the contribution of food groups to free sugar intake over time. *Sugar & sweets* and *juices*, were the main food sources of FS throughout the observation period (Figure 1), together contributing to more than half of FS intake. While *SSB*s were the third largest source for FS in the first 20 years of observation, *dairy products* have replaced *SSBs* in the last decades. In view of the fact that FS from *dairy products* was lower than FS from the other three investigated sources throughout the entire observation period, this is presumably due to the remarkably sharp decline in SSB intake (by over 50% between 1990 and 2016).

Our results for age trends in food group intake partly confirm the hypothesis of an age-depending shift from naturally occurring sugar sources such as fruits, milk, dairy products and juices, to those sources with high amounts of AS such as *sugar & sweets* and *SSB*, which was suggested from our previous trend analyses in total sugar and AS intake [30]. This shift is in accordance with results from other studies. Lytle et al. reported a change in dietary pattern in children between third and eight grade due to a decreased consumption of fruits, vegetables, and milk [57], whereas SSB consumption increased during puberty in several studies [57,58]. Daily SSB consumption in the second KiGGS survey was also higher among older as compared to younger children [54]. While age trends in FS intake from *SSB* and *juices* from the present analyses would support our initial hypothesis, age trends in FS intake especially from *sugar& sweets* are not in line with the hypothesis that intakes highest among 7/8 and 9/10 year-olds lower intakes among both younger children and adolescents (Figure 2). In addition, FS intake from *dairy products* decreased with age.

The observed shift in eating patterns may reflect the increasing autonomy in food choice with age. In addition, the environment of older children provides more food choices. Dairy products seem to be an exception, as they often contain both natural sugar and added sugar. Furthermore, marketing efforts explicitly focus on taste preferences of children and adolescents, which may have affected the intake of some food groups. In Germany, advertisement for SSB are more commonly directed at adolescents or young adults whereas advertisements for juices or some dairy products are targeted to younger children.

The knowledge of these age-dependent dietary patterns may contribute to the development of specific public health measures for children and adolescents.

Sex-specific differences in age trends emerged for FS intakes from *SSB* only. While FS intake in boys increased continuously with age, consumption in girls remained fairly stable throughout adolescence. This may to some extent reflect the commonly observed tendency of female adolescents to exhibit a more “healthy” food choice [59]. To our knowledge, to date, there are no data on sex- or gender-specific development of dietary patterns during childhood and adolescence, which could explain the difference in age trends observed in our study.

Age differences in FS intake from food groups were investigated only by one other study: Among European participants from the IDEFICS (Identification and prevention of Dietary- and lifestyle-induced health EFfects In Children and infantS) study, FS intake from dairy was significantly lower among older girls (6–<10 years) compared to younger ones (2–<6 years) [29], which supports our findings. In contrast to our results, FS intake from juices was significantly higher among older participants, but only among boys [29]. These differences most likely reflect differences in the methodology and/or characteristics of the study populations, such SES.

The relatively high SES of the DONALD study population may limit the generalizability of our results to the general German pediatric population [37]. Some studies showed a clear inverse association between SES and intake of sugary foods, especially SSB intake [60,61,62]. In the DONALD study, in particular, young children did not consume any SSB but instead consumed substantial amounts of fruit juices. Nevertheless, median total sugar and FS intake as well as absolute intake amounts of food groups observed in our sample are similar to intake levels reported from representative German nutrition surveys [63,64] or other European countries [65,66].

A further limitation of the current analyses is that we were not able to adjust for physical activity levels in the statistical model, as data on physical activity in the DONALD study has only been systematically collected since 2004.

The main strength of this study is its longitudinal design, allowing time and age trend analyses covering a period of 31 years in a large sample size using a large number of 3-day weighed dietary records. The weighed dietary records and the continuously updated in-house nutrient database LEBTAB allow the consideration of brand-specific sugar content in commercial products as well as sugars or sweetening agents such as syrups and honey, which are used for food preparation at home [39]. In addition, LEBTAB [39] accounts for changes in recipes over time and allows the estimation of both total FS intake and FS intake from different food groups. Finally, a variety of covariates that have been linked to dietary sugar intake could be considered as potential confounders [61,62,67].

## 5. Conclusions

Our study provides important insights into time and age trends in FS intake from different food groups relevant for future public health measures. While FS intake from *juices*, *SSB*, *sugar & sweets* and *dairy products* declined, especially in recent years, overall, FS intakes continue to exceed the recommendations of 10%E and therefore, call for further public health measures. Since the decline in FS intake from *sugar & sweets* was less pronounced and these products remained the main source for FS over the entire observation period, public health measures should not only focus on SSB, but also address a decline of FS intake from *sugar & sweets.* The consideration of age trends should also play a role in this context as primary school children particularly showed a high intake of FS from *sugar & sweets* and older children from *SSB*. Especially, since sugary foods are ubiquitously available and are offered in the daily environment of children and adolescents, e.g., in schools.

## Figures and Tables

**Table 1:** Classifications of the food groups.

Food Group	Components
*Sugar & sweets*	Sugars and other sweeteners (including syrups), sweet spreads, sweets and marshmallows, chocolate and bars, ice cream, jelly desserts, sweet sauces, sweet baking ingredients (e.g., marzipan)
*Dairy products*	unfermented (e.g., milk, cream, pudding) and fermented dairy products (e.g., yoghurt, buttermilk) all types of cheese (e.g., cream, soft, hard and processed cheese), dairy powder, vegan milk and cheese substitutes, instant milk beverages (e.g., cocoa)
*Fruits & vegetables*	Fresh, frozen, canned and dried fruits and vegetables
*Juices*	Fruits and vegetable juices, juice spritzers and smoothies
*Sugar sweetened beverages (SSB)*	Sweetened fruit juice drinks and nectars, soft drinks/sodas, sweetened teas and waters, instant beverages (except dairy drinks), sweetened sport drinks
*Sweet bread & cakes*	Sweet breads, pastries, cakes, pies, baking mixtures
*Ready to eat breakfast cereals (RTC)*	Ready to eat cereals and mueslis
*Others*	Eggs and egg meals (e.g., pancakes), meat and meat products, fish and fish products, vegetarian/vegan meat substitutes and spreads, fats and oils, flours, grains, breads, doughs, salty snacks, noodles, pasta, potatoes and potato products, nuts and seeds, legumes, alcoholic drinks, unsweetened teas, ready to eat meals, instant soups, sauces and dressings, spices, formula and baby food

**Table 2:** Participants’ overweight status and maternal characteristics of 1312 DONALD study participants.

Female	652 (49.7)
Anthropometrics	
Overweight ^1^	161 (12.3)
Maternal characteristics	
Overweight ^2^	432 (32.9)
High educational status ^3^	817 (62.3)
Employment	784 (59.8)

Values are frequencies (%); %E = percentage of total daily energy intake; ^1^ BMI cutoff values for children and adolescents [40]; ^2^ BMI > 25 kg/m^2^; ^3^ ≥12 years of schooling.

**Table 3:** Dietary characteristics from 10,761 dietary records of 1312 DONALD study participants (3–18 years) between 1985 and 2016, stratified by sex (*n* = 660 boys, *n* = 652 girls) and time periods.

	Girls			Boys		
	1985–1995	1996–2005	2006–2016	1985–1995	1996–2005	2006–2016
**n_records_**	1348	1977	1953	1375	1945	2163
**Age**	7.0 (4.7; 10.1)	9.0 (6.0; 13.0)	10.0 (6.0; 14.0)	7.0 (4.9; 10.3)	9.1 (6.0; 13.1)	9.3 (6.0; 14.0)
**TEI [kcal]**	1390 (1156; 1680)	1495 (1238; 1785)	1524 (1265; 1821)	1596 (1307; 1917)	1742 (1409; 2162)	1731 (1372; 2139)
TEI/BMR	1.4 (1.2; 1.6)	1.4 (1.2; 1.5)	1.4 (1.2; 1.5)	1.5 (1.3; 1.6)	1.4 (1.2; 1.6)	1.4 (1.2; 1.6)
**Carbohydrates [%E]**	49.8 (46.1; 53.5)	52.1 (48.3; 55.8)	52.3 (48.4; 56.3)	49.8 (46.0; 53.9)	51.9 (47.9; 55.9)	52.1 (48.1; 55.9)
**Total sugar [%E]**	27.5 (23.6; 31.7)	27.5 (23.1; 32.0)	25.5 (21.0; 30.6)	27.1 (23.2; 31.9)	27.6 (22.9; 32.1)	25.6 (21.0; 30.1)
**Added sugar [%E]**	12.5 (8.8; 16.4)	12.9 (9.4; 16.9)	11.8 (8.7; 15.5)	12.8 (9.3; 16.6)	13.0 (9.7; 17.3)	12.0 (8.9; 15.4)
**Free sugar intake [%E]**	16.5 (12.3; 20.6)	17.3 (13.2; 21.9)	16.0 (11.6; 20.4)	16.3 (12.3; 20.7)	17.9 (13.8; 22.4)	16.1 (12.1; 20.8)
FS from *sugar & sweets*	5.8 (3.7; 8.2)	5.5 (3.5; 8.0)	5.0 (3.0; 7.6)	5.5 (3.6; 7.8)	4.9 (2.9; 7.7)	4.7 (2.8; 7.2)
FS from *juices*	2.5 (0.0; 5.7)	3.2 (0.0; 6.5)	2.8 (0.0; 6.2)	2.1 (0.0; 5.1)	3.4 (0.7; 6.9)	2.9 (0.0; 6.2)
FS from *dairy products*	1.6 (0.4; 3.0)	1.6 (0.4; 2.9)	1.5 (0.3; 2.8)	1.6 (0.4; 3.0)	1.6 (0.4; 3.2)	1.5 (0.1; 3.1)
FS from *SSB*	1.9 (0.0; 5.0)	1.5 (0.0; 4.9)	0.0 (0.0; 3.3)	2.2 (0.0; 5.6)	2.1 (0.0; 4.9)	0.9 (0.0; 3.8)
FS from *sweet breads & cakes*	0.8 (0.1; 1.7)	0.9 (0.1; 2.0)	1.1 (0.2; 2.3)	0.7 (0.0; 1.8)	0.7 (0.0; 1.8)	1.0 (0.0; 2.3)
FS from *RTC*	0.0 (0.0; 0.3)	0.0 (0.0; 1.0)	0.0 (0.0; 0.8)	0.0 (0.0; 0.5)	0.0 (0.0; 1.5)	0.0 (0.0; 1.1)
FS from others	0.1 (0.0; 0.3)	0.2 (0.1; 0.5)	0.4 (0.2; 0.8)	0.1 (0.0; 0.3)	0.2 (0.0; 0.5)	0.4 (0.2; 0.8)
FS from *fruits & vegetables*	0.0 (0.0; 0.2)	0.0 (0.0; 0.0)	0.0 (0.0; 0.0)	0.0 (0.0; 0.2)	0.0 (0.0; 0.0)	0.0 (0.0; 0.0)

Values are medians (25th, 75th percentile); n_records_ = number of records; TEI total energy intake; TEI/BMR total energy intake/basal metabolic rate; FS = Free sugar; %E = Percentage of total daily energy intake, RTC = Ready-to-eat cereals.

**Table 4:** Dietary characteristics from 10,761 dietary records of 1312 DONALD study participants (3–18 years) between 1985 and 2016, stratified by sex (*n* = 660 boys, *n* = 652 girls) and age groups.

	Girls				Boys			
	3–5 Years	6–10 Years	11–14 Years	15–18 Years	3–5 Years	6–10 Years	11–14 Years	15–18 Years
**n_records_**	1382	1895	1163	838	1428	1978	1216	861
**TEI [kcal]**	1133	1497	1772	1771	1242	1673	2010	2452
	(1003; 1274)	(1309; 1685)	(1529; 2021)	(1498; 2056)	(1091; 1402)	(1466; 1884)	(1743; 2319)	(2100; 2819)
TEI/BMR	1.36 (1.23; 1.51)	1.43 (1.28;1.59)	1.35 (1.15; 1.52)	1.19 (1.00; 1.40)	1.39 (1.24; 1.54)	1.49 (1.32; 1.64)	1.36 (1.17; 1.54)	1.31 (1.11; 1.51)
**Carbohydrates [%E]**	51.4 (47.4; 55.3)	51.8 (48.2; 55.7)	51.4 (47.5; 55.5)	51.4 (47.2; 55.6)	51.8 (47.9; 56.1)	51.8 (47.9; 55.6)	51.3 (47.6; 55.1)	50.1 (45.9; 54.5)
**Total sugar [%E]**	28.1 (24.0; 32.7)	27.4 (23.3; 31.6)	25.6 (21.3; 30.3)	25.0 (19.8; 30.0)	28.5 (24.4; 33.5)	26.9 (23.0; 31.2)	25.7 (21.3; 30.3)	23.5 (19.5; 28.7)
**Added sugar [%E]**	11.6 (8.6; 14.9)	13.3 (9.9; 16.8)	12.8 (8.9; 15.6)	11.6 (7.9; 15.8)	11.7 (8.5; 15.3)	13.0 (9.7; 16.6)	13.2 (9.7; 17.2)	12.2 (8.5; 16.5)
**Free sugar intake [%E]**	16.3 (12.1; 20.6)	17.5 (13.4; 21.5)	16.7 (12.4; 21.3)	15.2 (10.8; 20.0)	16.9 (12.6; 21.7)	17.0 (13.1; 21.4)	16.9 (13.0; 21.4)	15.8 (11.7; 20.7)
FS from *sugar & sweets*	5.4 (3.4; 7.9)	6.0 (3.9; 8.6)	5.3 (3.3; 7.9)	4.0 (2.2; 6.5)	5.2 (3.3; 7.6)	5.5 (3.5; 7.8)	4.9 (3.0; 7.6)	3.8 (1.8; 6.1)
FS from *juices*	3.6 (0.7; 7.1)	3.0 (0.0; 6.4)	2.4 (0.0; 5.5)	1.8 (0.0; 5.2)	4.0 (1.1, 7.6)	2.8 (0.0; 6.2)	2.2 (0.0; 5.2)	2.2 (0.0; 5.2)
FS from *dairy products*	1.8 (0.5; 3.3)	1.7 (0.6; 3.1)	1.4 (0.2; 2.6)	1.1 (0.0; 2.4)	1.8 (0.5; 3.4)	1.7 (0.5; 3.3)	1.4 (0.2; 2.8)	1.0 (0.0; 2.5)
FS from *SSB*	0.0 (0.0; 2.7)	1.2 (0.0; 4.1)	1.9 (0.0; 5.6)	1.9 (0.0; 5.9)	0.0 (0.0; 2.8)	1.6 (0.0; 4.4)	2.5 (0.0; 6.5)	3.2 (0.0; 7.6)
FS from *sweet breads & cakes*	1.1 (0.3; 2.2)	1.1 (0.2; 2.2)	0.8 (0.0; 1.9)	0.7 (0.0; 1.9)	1.0 (0.0; 2.2)	1.0 (0.1; 2.2)	0.6 (0.0; 1.8)	0.3 (0.0; 1.4)
FS from *RTC*	0.0 (0.0; 0.5)	0.0 (0.0; 0.9)	0.0 (0.0; 0.9)	0.0 (0.0; 0.9)	0.0 (0.0; 0.6)	0.1 (0.0; 1.4)	0.0 (0.0; 1.3)	0.0 (0.0; 1.1)
FS from others	0.2 (0.0; 0.4)	0.2 (0.0; 0.5)	0.3 (0.1; 0.7)	0.4 (0.1; 0.8)	0.2 (0.0; 0.5)	0.2 (0.1; 0.6)	0.3 (0.1; 0.7)	0.4 (0.1; 0.9)
FS from *fruits & vegetables*	0.0 (0.0; 0.0)	0.0 (0.0; 0.0)	0.0 (0.0; 0.0)	0.0 (0.0; 0.0)	0.0 (0.0; 0.0)	0.0 (0.0; 0.0)	0.0 (0.0; 0.0)	0.0 (0.0; 0.0)

Values are medians (25th, 75th percentile); n_records_ = number of records; TEI total energy intake; TEI/BMR total energy intake/basal metabolic rate; FS = Free sugar; %E = Percentage of total daily energy intake, RTC = Ready to eat cereals.

**Table 5:** Time and age trends in free sugar intake from different food groups of 10,761 dietary records of 1312 DONALD study participants (*n* = 660 boys, *n* = 652 girls) (3–18 years) between 1985 and 2016.

	Age Trend Per Year of Age (3–18 Years) ^a^			Time Trend Per Study Year (1985–2016) ^b^			Interaction of Time and Age
	Age β (*p*)	Age^2^ β (*p*)	Age^3^ β (*p*)	Time β (*p*)	Time^2^ β (*p*)	Time^3^ β (*p*)	Age × Time β (*p*)
**FS from *sugar & sweets***							
Girls ^c^ Unadjusted model	0.9268 (<0.0001)	−0.07895 (<0.0001)	0.001717 (0.0029)	−0.04032 (<0.0001)			
Adjusted model	0.9330 **(<0.0001)**	−0.07851 **(<0.0001)**	0.001697 **(0.0033)**	−0.04294 **(<0.0001)**			
Boys ^d^ Unadjusted model	0.3994 (<0.0001)	−0.02390 (<0.0001)		0.01535 (0.8293)	−0.00605 (0.2198)	0.000156 (0.1144)	
Adjusted model	0.4158 **(<0.0001)**	−0.02409 **(<0.0001)**		0.01052 (0.8833)	−0.00548 (0.2700)	0.000144 (0.1482)	
**FS from *juices*** ^e^							
Unadjusted model	−0.7443 (0.0001)	0.05349 (0.0009)	−0.00136 (0.0084)	0.3250 (<0.0001)	−0.00895 (<0.0001)		
Adjusted model	−0.7304 **(0.0001)**	0.05339 **(0.0009)**	−0.00136 **(0.0082)**	0.3150 **(<0.0001)**	−0.00892 **(<0.0001)**		
**FS from *dairy products*** ^f^							
Unadjusted model	0.1192 (0.0699)	−0.01531 (0.0273)	0.000471 (0.0352)	−0.03703 (0.1907)	0.006421 (0.0009)	−0.00017 (<0.0001)	−0.00149 (0.0489)
Adjusted model	0.1159 (0.0780)	−0.01604 **(0.0207)**	0.000492 **(0.0280)**	−0.02873 (0.3094)	0.006435 **(0.0009)**	−0.00017 **(<0.0001)**	−0.00140 (0.0634)
**FS from *SSB***							
Girls ^g^ Unadjusted model	−0.04422 (0.8096)	0.03543 (0.0715)	−0.00144 (0.0241)	−0.4051 (<0.0001)	0.02430 (<0.0001)	−0.00049 (<0.0001)	
Adjusted model	−0.07104 (0.6987)	0.03659 (0.0629)	−0.00148 **(0.0211)**	−0.3789 **(<0.0001)**	0.02412 **(<0.0001)**	−0.00049 **(<0.0001)**	
Boys ^h^ Unadjusted model	0.2649 (<0.0001)			−0.2793 (0.0004)	0.01462 (0.0085)	−0.00030 (0.0072)	
Adjusted model	0.2433 **(<0.0001)**			−0.2359 **(0.0028)**	0.01329 **(0.0165)**	−0.00028 **(0.0135)**	

Time and age trends were tested using polynomial mixed-effects regression models; significant *p*-values of the adjusted models are marked bold; FS = Free sugar; *SSB* = *Sugar sweetened beverages*; ^a^ age = linear age trend, age^2^ = quadratic age trend, age^3^ = cubic age trend, ^b^ time = linear time trend, time^2^ = quadratic time trend, time^3^ = cubic time trend; ^c^ Model contains a random statement for the family level with an unstructured covariance structure and a random statement for the person level with an unstructured covariance structure. Adjusted for number of weekdays per record (1/2/3) and overweight status (yes/no); ^d^ Model contains a random statement for the family level with an unstructured covariance structure and a random statement for the person level with an unstructured covariance structure. Adjusted for overweight status (yes/no), number of weekdays per record (1/2/3), maternal employment (yes/no), high maternal educational status (yes/no); ^e^ Model contains a repeated statement with a heterogeneous Toeplitz covariance structure. Adjusted for overweight status (yes/no), high maternal educational status (yes/no); ^f^ Model contains a random statement for the family level with an unstructured covariance structure and a random statement for the person level with an unstructured covariance structure. Adjusted for high maternal educational status (yes/no), number of weekdays per record (1/2/3); ^g^ Model contains a random statement for the family level with an unstructured covariance structure and a random statement for the person level with an unstructured covariance structure. Adjusted for high maternal educational status (yes/no), number of weekdays per record (1/2/3); ^h^ Model contains a repeated statement with a heterogeneous Toeplitz covariance structure. Adjusted for high maternal educational status (yes/no).

## Supplementary Materials

The following are available online at https://www.mdpi.com/2072-6643/12/1/20/s1, Figure S1: Time and age trends in FS intake from sweet bread & cakes, Figure S2: Time and age trends in FS intake from RTC, Figure S3: Time and age trends in FS intake from others, Table S1: Time and age trends in free sugar intake from different food groups of 10,761 dietary records of 1312 DONALD study participants (*n* = 660 boys, *n* = 652 girls) (3–18 years) between 1985 and 2016.
