# Supplementary material for: Time and Age Trends in Free Sugar Intake from Food Groups among Children and Adolescents between 1985 and 2016
Source: Nutrients. 2019 Dec 20;12(1):20. doi: 10.3390/nu12010020 (PMC7019792; doi:10.3390/nu12010020)
Supplement: Supplementary file 1 [file nutrients-12-00020-s001.pdf]

nutrients

MDPI

Table S1: Time and age trends in free sugar intake from different food groups of 10,761 dietary records of 1,312 DONALD study participants (n = 660 boys, n = 652 girls) (3–18 years) between 1985 and 2016

	Age trend per year of age (3-18 years)[a]			Time trend per study year (1985-2016)[b]			Interaction of time and age
	Age β (p)	Age^2 β (p)	Age^3 β (p)	Time β (p)	Time^2 β (p)	Time^3 β (p)	Age x Time β (p)
**FS from *sweet bread & cakes***							
Boys[c]							
Unadjusted model	0.1573 (0.0252)	-0.01782 (0.0180)	0.000460 (0.0586)	-0.06815 (0.0136)	0.005533 (0.0038)	-0.00010 (0.0087)	
Adjusted model	0.1517 (0.0305)	-0.01680 (0.0254)	0.000430 (0.0767)	-0.07273 (0.0084)	0.005750 (0.0026)	-0.00011 (0.0061)	
Girls[d]							
Unadjusted model	0.2285 (0.0017)	-0.02813 (0.0003)	0.000893 (0.0004)	-0.07492 (0.0062)	0.006724 (0.0004)	-0.00013 (0.0006)	
Adjusted model	0.2191 (0.0025)	-0.02665 (0.0006)	0.000849 (0.0007)	-0.07397 (0.0067)	0.006537 (0.0006)	-0.00013 (0.0008)	
**FS from *RTC***							
Boys[e]							
Unadjusted model	0.3022 (<0.0001)	-0.02523 (0.0004)	0.000638 (0.0063)	0.1110 (<0.0001)	-0.00495 (0.0045)	0.000063 (0.0792)	
Adjusted model	0.3116 (<0.0001)	-0.02649 (0.0002)	0.000677 (0.0036)	0.1122 (<0.0001)	-0.00495 (0.0045)	0.000063 (0.0780)	
Girls[f]							
Unadjusted model	0.2673 (<0.0001)	-0.02361 (<0.0001)	0.000580 (0.0030)	0.04111 (<0.0001)	-0.00127 (<0.0001)		0.001045 (0.0359)
Adjusted model	0.2796 (<0.0001)	-0.02507 (<0.0001)	0.000626 (0.0013)	0.04267 (<0.0001)	-0.00124 (<0.0001)		0.000917 (0.0661)
**FS from *other sources*[g]**							
Unadjusted model	0.003417 (0.9072)	-0.00280 (0.3713)	0.000138 (0.1773)	-0.03480 (0.0012)	0.002869 (<0.0001)	-0.00006 (<0.0001)	0.001437 (<0.0001)
Adjusted model	-0.00328 (0.9111)	-0.00229 (0.4644)	0.000123 (0.2296)	-0.03272 (0.0025)	0.002711 (0.0003)	-0.00006 (0.0002)	0.001512 (<0.0001)

Time and age trends were tested using polynomial mixed-effects regression models
FS=Free sugar
[a]Age=linear age trend, age^2=quadratic age trend, age^3=cubic age trend [b]time=linear time trend, time^2=quadratic time trend, time^3=cubic time trend
[c]Model contains a random statement for the family level with an unstructured covariance structure. Adjusted for number of weekdays per record (1/2/3).
[d]Model contains a random statement for the family level with factor analytic covariance structure and a random statement for the person level with an unstructured covariance structure. Adjusted for number of weekdays per record (1/2/3).
[e]Model contains a repeated statement with a heterogeneous toeplitz covariance structure. Adjusted for number of weekdays per record (1/2/3).

[f]Model contains a repeated statement with a factor analytic covariance structure. Adjusted for number of weekdays per record (1/2/3).

[g]Model contains a repeated statement with a factor analytic covariance structure. Adjusted for number of weekdays per record (1/2/3), high maternal educational status (yes/no), overweight status (yes/no), maternal employment (yes/no).

.

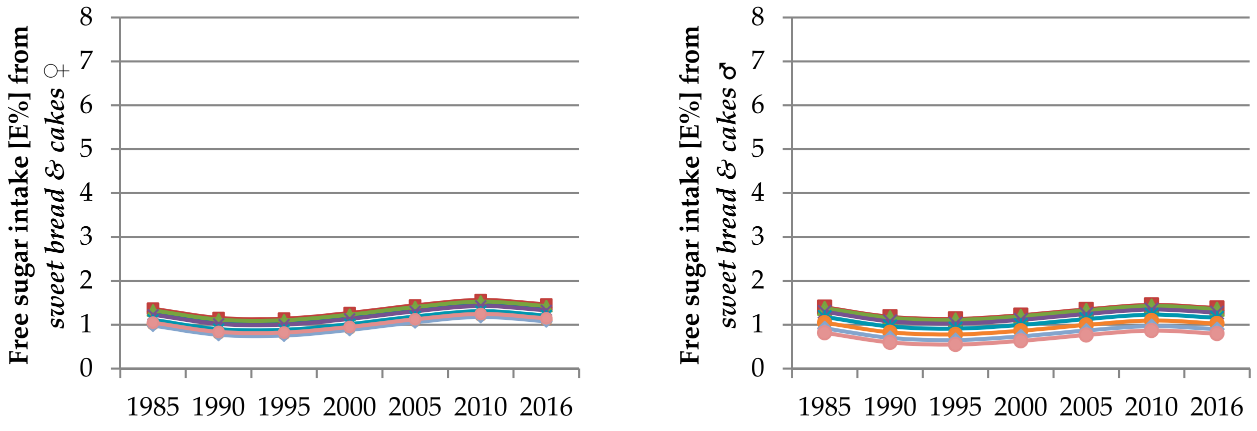

a b

Figure S1. Time and age trends in FS intake from sweet bread & cakes among girls (a) and boys (b) of 10,761 dietary records of 660 male and 652 female DONALD study participants (3-18 years) between 1985 and 2016, predicted by polynomial mixed-effects regression models (see Table S1) (blue rhombi 3/4-year-olds, red squares 5/6-year-olds, green triangles 7/8-year-olds, purple crosses 9/10-year-olds, turquoise stars 11/12-year-olds, orange circles 13/14-year-olds, light blue rhombi 15/16-year-olds, pink circles 17/18-year-olds).

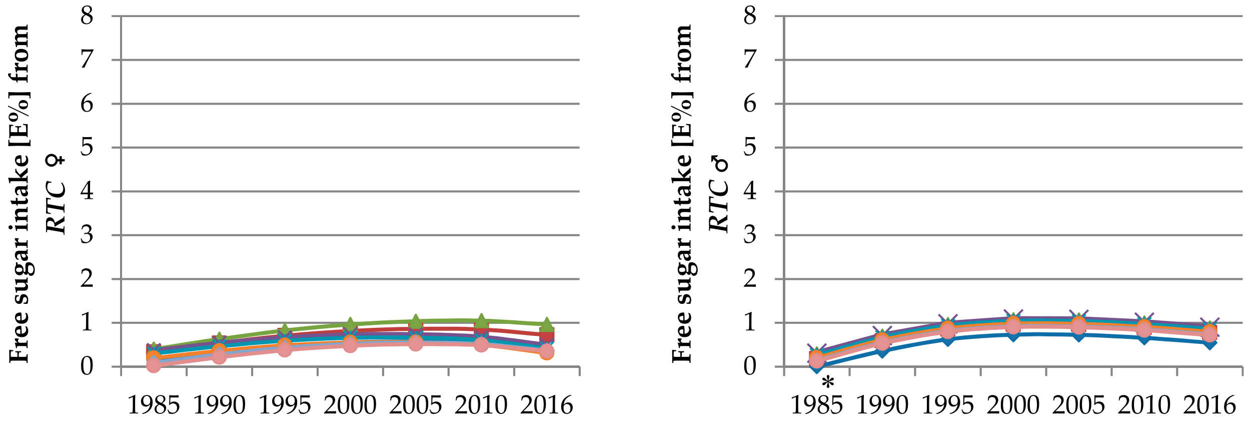

a b

Figure S2. Time and age trends in FS intake from *RTC* among girls (a) and boys (b) of 10,761 dietary records of 660 male and 652 female DONALD study participants (3-18 years) between 1985 and 2016, predicted by polynomial mixed-effects regression models (see Table S1) (blue rhombi 3/4-year-olds, red squares 5/6-year-olds, green triangles 7/8-year-olds, purple crosses 9/10-year-olds, turquoise stars 11/12-year-olds, orange circles 13/14-year-olds, light blue rhombi 15/16-year-olds, pink circles 17/18-year-olds).

*** FS intake from RTC among 3/4 year olds in 1985 was set to 0, ifs the calculation of predicted values from the regression equations reveals values <0.**

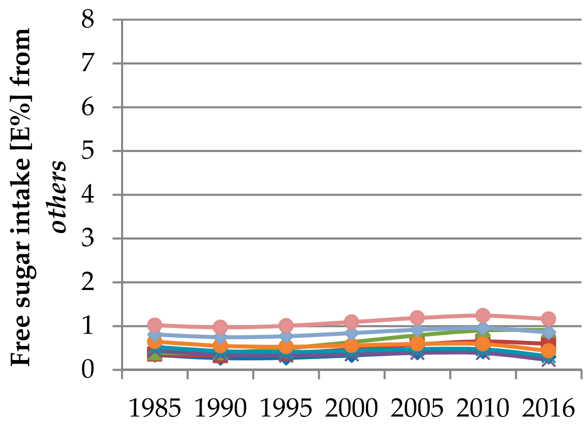

Figure S3. Time and age trends in FS intake from *others* of 10,761 dietary records of 1,312 DONALD study participants (3-18 years) between 1985 and 2016, predicted by polynomial mixed-effects regression models (see Table S1) (blue rhombi 3/4-year-olds, red squares 5/6-year-olds, green triangles 7/8-year-olds, purple crosses 9/10-year-olds, turquoise stars 11/12-year-olds, orange circles 13/14-year-olds, light blue rhombi 15/16-year-olds, pink circles 17/18-year-olds).
